# Identification of QTNs, QTN-by-environment interactions for plant height and ear height in maize multi-environment GWAS

**DOI:** 10.3389/fpls.2023.1284403

**Published:** 2023-11-29

**Authors:** Guoping Shu, Aifang Wang, Xingchuan Wang, Ruijie Chen, Fei Gao, Aifen Wang, Ting Li, Yibo Wang

**Affiliations:** ^1^Center of Biotechnology, Beijing Lantron Seed, LongPing High-tech Corp., Zhengzhou, Henan, China; ^2^2Experiment Station, Henan LongPing-Lantron AgriScience and Technology Co., LTD, Zhengzhou, Henan, China; ^3^LongPing High-tech Corp., Zhengzhou, Henan, China

**Keywords:** maize, multi-environment-GWAS, plant height, ear height, QTN, QTN-by-Environment interaction (QEI)

## Abstract

Plant height (PH) and ear height (EH) are important traits associated with biomass, lodging resistance, and grain yield in maize. There were strong effects of genotype x environment interaction (GEI) on plant height and ear height of maize. In this study, 203 maize inbred lines were grown at five locations across China’s Spring and Summer corn belts, and plant height (PH) and ear height (EH) phenotype data were collected and grouped using GGE biplot. Five locations fell into two distinct groups (or mega environments) that coincide with two corn ecological zones called Summer Corn Belt and Spring Corn Belt. In total, 73,174 SNPs collected using GBS sequencing platform were used as genotype data and a recently released multi-environment GWAS software package IIIVmrMLM was employed to identify QTNs and QTN x environment (corn belt) interaction (QEIs); 12 and 11 statistically significant QEIs for PH and EH were detected respectively and their phenotypic effects were further partitioned into Add*E and Dom*E components. There were 28 and 25 corn-belt-specific QTNs for PH and EH identified, respectively. The result shows that there are a large number of genetic loci underlying the PH and EH GEIs and IIIVmrMLM is a powerful tool in discovering QTNs that have significant QTN-by-Environment interaction. PH and EH candidate genes were annotated based on transcriptomic analysis and haplotype analysis. EH related-QEI *S10_135* (*Zm00001d025947*, *saur76*, small auxin up RNA76) and PH related-QEI *S4_4* (*Zm00001d049692*, *mads32*, encoding MADS-transcription factor 32), and corn-belt specific QTNs including *S10_4* (*Zm00001d023333*, *sdg127*, set domain gene127) and *S7_1* (*Zm00001d018614*, *GLR3.4*, and glutamate receptor 3.4 or *Zm00001d018616*, DDRGK domain-containing protein) were reported, and the relationship among GEIs, QEIs and phenotypic plasticity and their biological and breeding implications were discussed.

## Introduction

Maize is a cereal plant of the grass family (*Poaceae*) and its domesticated form, the grain corn, is one of the most important crop for food, feed, energy, and industrial materials in the world. China is the second largest grain corn producer after USA and Summer corn belt (33%) and Spring corn belt (47%) are ecological regions that contribute 80% of China’s total corn grain output (Shu et al., 2021; Dai et al., 2010). Plant height and ear height are two important maize traits that affect biomass, lodging resistance, and corn grain yield. Enhancing yield and yield stability through genetically controlling plant height and ear height have been important goals in maize genetics and corn breeding. A large number of QTL and QTN loci in maize that associated with plant height and ear height have been identified and reported by quantitative trait loci (QTL) mapping and genome-wide association studies (GWAS) and verified by genetic fine mapping, transcriptomic analyses, and functional genetic analysis (Bai et al., 2010; Zhang et al., 2011; Li et al., 2016; Zheng et al., 2016; Ding et al., 2017; Si et al., 2020; Wang et al., 2023; Jin et al., 2023; Napier et al., 2023; Zhou et al., 2023); among them, *Dwarf 8, Dwarf 9* encodes maize DELLA proteins (Lawit et al., 2010), *Ga3ox2* encodes a GA3 b-hydroxylase (Teng et al., 2013), *ZmTE1*, likely regulates auxin signaling, cell division, and cell elongation (Wang et al., 2022a), *ZmRPH1* that regulate both plant height and ear height, encodes a microtubule-associated protein (Li et al., 2020), *ZmDLE1* is associated with a candidate gene that effectively regulate maize plant height and ear height (Zhou et al., 2023), and a set of growth regulating factors genes (*ZmGRF*) that co-express with a large set of plant height and ear height loci (Si et al., 2020). In the classic Brachytic2 locus (Multani et al., 2003), a number of different alleles or genetic variants have been reported that show various degree of phenotype effect on plant height and ear height and that differentially regulate downstream genes involved in gibberellin and brassinosteroid biosynthesis, auxin transport and cellulose synthesis (Xing et al., 2015; Wei et al., 2018).

Phenotypic plasticity is the property of a given genotype to produce different phenotypes in response to distinct environmental conditions (Pigliucci, 2001) or the ability of a single genotype to produce different phenotypes in response to environmental stimuli (Napier et al., 2023) and it is a joint result of overall environmental effect and genetic effects across environments (Li et al., 2018; Liu et al., 2020b). Genotype x Environment Interaction (GEI) is a special case of environmental plasticity where the two genotypes respond in opposite directions to the changes in the environment (Mather and Caligari, 1974; Laitinen and Nikoloski, 2019). Genotype x Environment Interaction (GEI) on corn yield and agronomic traits has been a major goal of the USA Maize Genomes to Fields Initiative (Alkhalifah et al., 2018; Rogers et al., 2021). Phenotypic plasticity and GEI in maize and other crops have been well-known in plant height and ear height (Wallace et al., 2016; Perrier et al., 2017; Mu et al., 2022). Some environmental factors, such as the difference between day and night temperature (also referred to as DIF) have been shown to influence internode length and plant height (Myster and Moe, 1995). Corn inbred lines with tropical germplasm introgression have been shown to respond to daylength or photoperiod (Coles et al., 2010; Lin et al., 2021; Su et al., 2021; Fei et al., 2022; Osnato et al., 2022). Explaining and predicting phenotypes requires the holistic examination of genomes, environments, and their interaction throughout the spatial and temporal dimensions of an organism’s life cycle (Li et al., 2021; Schneider, 2022). In traditional G x E studies, a genotype is treated as a black box of the entire genome, and various statistical models were developed to understand the pattern and mechanism of GEI (Mather and Caligari, 1974; Shu and Fan, 1986; Cooper and DeLacy, 1994; Malosetti et al., 2013). Further partitioning Genome x Environmental interaction or GEI into QTN x E (QEI) or Gene x E (GEI)) is a breakthrough and only becomes feasible in recent years with the availability of whole genome sequencing technology, transcriptomic technology, the availability of abundant DNA polymorphic markers such as SNP and SSR, and improved GWAS methodologies (Xiao et al., 2017; Laitinen and Nikoloski, 2019; Li et al., 2022a; Li et al., 2022b; Jin et al., 2023; Napier et al., 2023).

In this study, we have conducted a multi-environment GWAS using the newly released GWAS software package developed by Li et al. (2022a); Li et al. (2022b) called IIIVmrMLM with the objective of detecting QEIs and QTNs, and estimating their additive-by-environment (add*E) and dominance-by-environment (dom*E) interaction effects of QEIs, and additive effects(add) and dominant effects(dom) of corn-belt specific QTNs. Candidate genes in the surrounding chromosomal regions of these QEIs and QTNs are mined and verified by transcriptomic analysis and haplotype analysis, and their implications to understanding the GEI, and phenotypic plasticity of PH and EH were discussed.

## Materials and methods

### Germplasm and phenotype evaluation

A diversity panel of 490 inbred lines from Shu et al. (2021) was used for this study, 203 inbred lines (accessions) that grow and seed well in both the Summer Corn Belt and Spring Corn Belt were elected for phenotyping in 2013. Five locations or environments with different latitudes across the Summer and Spring Corn Belt that produce over 80% of China’s grain corn were selected for phenotyping, which include a location at the southern end of the Summer Corn Belt, Dancheng (DC, latitude 33.645°N, and longitude 115.177°E) and a location at the northern end of China’s Spring Corn Belt, Binxian (BX, latitude 45.759°N, and longitude 127.486°E), and three locations in between: Zhengzhou (ZZ, latitude 34.859°N, and longitude 113.368°E, Summer Corn Belt), Ningjin (NJ, latitude 37.652°N, and longitude 116.800°E, Summer Corn Belt), and Tieling (TL, latitude 42.547°N, and longitude 124.159°E, Spring Corn Belt). At all five locations, the same set of 203 inbreds were planted in the same three-row plots in a complete randomized design (Niu et al., 2013) and five individuals were randomly sampled from each plot to measure plant height and ear height.

### Phenotype and environment analysis

The mean values of each inbred for PH and EH in each location (**Table S1**) were used in the summary statistics, correlation analysis, GGE biplot, and Two-way ANOVA. Summary statistics were obtained by R package ‘pastecs’, and correlation analysis and plots between different environments for plant height and ear height were completed by R package ‘PerformanceAnalytics’. Mega-environments were identified by GGE biplot using the GGEBiplotGUI_1.0-9 package (Frutos et al., 2014) in RStudio software (RStudio, PBC, Boston, MA, USA). Relationships between PH and EH in each location were examined using Pearson correlation coefficients by R. The mean values of plant height and ear height in each mega-environment group were used as phenotype values to identify the significant QTN-by-environment interactions (QEIs). Two-way ANOVA was carried out using the SAS 9.3 (SAS Institute Inc., Cary, NC, USA).

### DNA sequencing, genotyping, linkage disequilibrium and population structure

Leaf sample from each inbred line was used for DNA extraction with a CTAB procedure. DNA sequencing follows a protocol of Elshire et al. (2011). Genomic DNA was digested with the restriction enzyme ApeK1. Genotyping-by-Sequencing or GBS libraries were constructed in 96-plex and sequenced on Illumina HiSeq 2000. SNP calling was performed using the TASSEL-GBS pipeline (Glaubitz et al., 2014) and B73 RefGen V2.0 as the reference genome. Initially, 876,297 SNP was filtered with minor allele frequency (MAF) > 5%, missing rate < 20% (Shu et al., 2021; Shu et al., 2023), and data for 73,174 high-quality SNP loci was kept for genome-wide association studies (GWAS). Minor allele frequency (MAF) and proportion heterozygous of filtered SNPs (73,174 SNPs) was calculated by TASSEL 5.2.25. The percentage of SNP with different Minor allele frequency (MAF) and proportion heterozygous was counted and shown in a bar chart (**Figure S1**).

Linkage disequilibrium (LD) analysis was carried out by TASSEL 5.2.25 (https://www.maizegenetics.net/tassel, Bradbury et al., 2007) with LD window size 50 for all filtered SNP on each chromosome. Structure 2.3.4 (Hubisz et al., 2009) was used to detect the population structure among all 203 maize inbred lines using 7296 Tag-SNP extracted from 73175 SNPs by Haploview 4.2 (Barrett et al., 2005). Burn-in period and Monte Carlo Markov Chain (MCMC) replication number were set as 5,000 and 50,000 respectively for each run. Seven independent runs were performed with subpopulation number k= 3 to 9. The delta K values were estimated and output by Structure 2.3.4.

### Genome wide association studies by IIIVmrMLM

IIIVmrMLM, A software package that implements the 3VmrMLM model (Li et al., 2022a; Li et al., 2022b) was employed for genome-wide association studies (GWAS). In the single-locus module, 3VmrMLM includes two steps: 1) genome-scanning was employed, and SNP loci that were significant (p < 0.01) in Wald test were kept for the following analysis. A midresult file is output after step 1; 2) all the loci identified in step 1 were incorporated into the Multi-locus Model, all the effects were estimated by empirical Bayes, and the loci with LOD score larger than 3.0 of likelihood ratio test were outputted.

In this study, 73,174 filtered SNPs were used as genotype data, the Q matrix was calculated by the Structure 2.3.4 software under the best K value, the parameter “method” was set to “Multi_env” mode, other parameters were set as default values. The critical P-value and LOD score were set as 0.05/m and 3.0, respectively, for significant and suggested QTNs and QEIs, where m is the number of markers (Li et al., 2022b).

To identify QEIs, the phenotype data from five locations were grouped into the summer corn belt group (E1) containing data from three locations (Dancheng, Zhengzhou, Ningjin) and the spring corn belt group (E2, containing data from Tieling and Binxian), the mean value of all locations within each corn-belt group was calculated for each genotype and used as input data to IIIVmrMLM software under “Multi_env” module. The additive-by-environment (add*E) and dominance-by-environment (dom*E) interaction effects of QEIs were estimated and outputted in the final result.

To identify summer corn belt specific QTNs, the trait phenotype data of a genotype from three locations within the Summer Corn Belt was used, and the phenotype value at each location was used as input data for the IIIVmrMLM software under “Multi_env” module. Similarly, phenotype data from two locations within the spring corn belt was used to identify spring corn belt specific QTNs. The additive effects(add) and dominant effects(dom) of corn-belt specific QTNs were estimated and outputted in the final results of Summer and Spring Corn Belt.

### Candidate gene annotations of QEIs and QTNs, and patterns of QTN x E interaction

The fasta sequences containing significant QEIs and QTNs identified by IIIVmrMLM were re-aligned to the B73 v4 reference genome using NCBI BLAST-2.12.0+ (Camacho et al., 2009) to obtain a more accurate physical position for better gene annotations (https://www.maizegdb.org/gbrowse). To identify candidate genes that are associated with a QEI or QTN, we first conducted a primary screening within the chromosomal region 100kb up and down the significant QEI or QTN, then software ANOVAR was used for further screening; ANOVAR only output a candidate that meets the following criteria: the significant QTN or QEI is located within the transcriptional sequence of the candidate (further categorized as in Exon (synonymous or non-synonymous), Intron,3′-UTR, and 5′-UTR or within 1kb upstream or downstream of the candidate. The patterns of key QEIs were visualized by line chart.

### Candidate gene identification and tissue-specific expression analysis

The polymorphic SNPs surrounding key significant QEIs and QTNs and their PH and EH phenotype association from the midresult file and the relationship between SNPs and gene structures was studied using scatter and gene structure diagram. For each candidate gene, transcriptomic databases at MaizeGDB (MaizeGDB, https://www.maizegdb.org/) were searched for its expression profiles in different organs and tissues across different developmental stages. Haplotype analysis was used to verify the phenotype effect of important QTNs.

## Results

### Phenotypic analyses and mega-environment grouping

The descriptive statistics for PH and EH at five locations or growth environments are presented in **Table 1**. Variation of PH, measured by CV ranges from 12% to 15% within each location. The range and the degree of variation in PH in the Spring Corn Belts is larger than in the Summer Corn Belt. The absolute values of kurtosis and skewness were all less than 1 (**Table 1**), indicating that the phenotype data do not significantly depart from a normal distribution and are suitable for GWAS. Variation of EH measured by CV ranges from 19.3% to 29.6% within each location, Which is larger than PH. The range of variation in EH in the Spring Corn Belt is much larger than in the Summer Corn Belts.

**Table 1:** Descriptive statistics for PH, EH among 203 accessions across five environments.

Traits	Corn belt	Environments	Latitude	No. of Inbreds	Max.-Min. (cm)	Mean ± SD	CV (%)	Skewness	Kurtosis	CC(with EH)
PH	E1	DC	33.6° N	202	110-241	178.0 ± 21.4	12	0.13	0.14	0.56***
		ZZ	34.9° N	203	113.8-263.6	174.3 ± 25.1	14.4	0.49	0.42	0.75***
		NJ	37.7° N	201	115-250	184.6 ± 24.3	13.2	0.37	0.04	0.65***
	E2	TL	42.5° N	203	149-290	209.3 ± 29.8	14.2	0.23	-0.46	0.65***
		BX	45.8° N	202	89-245	169.6 ± 25.4	15	-0.16	0.07	0.51***
EH	E1	DC	33.6° N	202	32-109	66.8 ± 13.8	20.7	0.01	-0.29	
		ZZ	34.9° N	203	30.4-115.8	68.7 ± 13.3	19.3	-0.06	0.35	
		NJ	37.7° N	201	40-110	74.3 ± 14.4	19.3	0.21	-0.06	
	E2	TL	42.5° N	203	43-130	85.1 ± 18.7	22	0.06	-0.7	
		BX	45.8° N	201	23.5-114.1	60.2 ± 17.8	29.6	0.21	-0.14	

DC, Dancheng; ZZ, Zhengzhou; NJ, Ningjin; TL, Tieling; BX, Binxian. CC, Correlation coefficient. ***P < 0.001.

**Figure 1:**
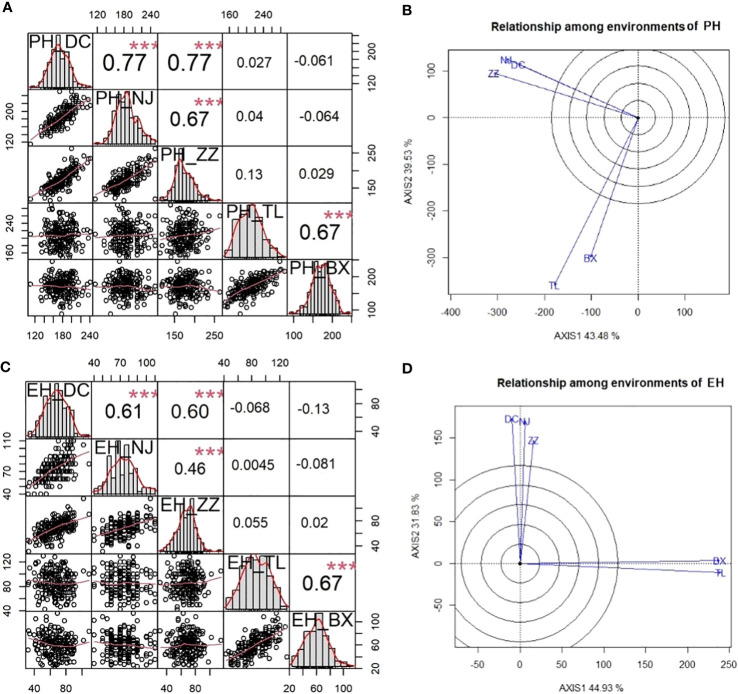
Phenotypic correlations between five environments within and between two corn belts viewed by correlation matrix and GGE biplot for PH and EH. **(A)** and **(C)** are correlation matrix among five environments for PH **(A)** and EH **(C)**; **(B)** and **(D)** are GGE biplots for PH **(B)** and EH **(D)**. ***P < 0.001.

The phenotypic correlation between each environment-pair for PH and EH among three Summer Corn Belt locations [Dancheng (DC), Zhengzhou (ZZ), and Ningjin (NJ)] and between two Spring Corn Belt locations Tieling (TL) and Binxian (BX), are shown in **Figures 1A and C**. As the scatter plot and correlation coefficients in **Figure 1A** show, the within-corn belt location-pair correlation coefficients (PH*PH) are 0.77, 0.77, and 0.66 for three Summer Corn Belt locations and 0.66 for two Spring Corn Belt locations for PH, which are significant at 0.01 level. Whereas, the six between-corn belt correlation coefficients are from 0.03 to 0.12, which are not significant at the 0.05 level. The same pattern was observed for EH (**Figure 1C**), suggesting a high location-location correlation within each corn belt and nearly zero location-location correlation between the two corn belts. The lack of phenotypic correlation between the two corn belts was also revealed by biplot for PH (**Figure 1B**) and EH (**Figure 1D**), which shows that the location vectors within the same corn belts form tight bundles, and the two vector bundles form a nearly vertical angle. Thus, GGE biplot groups the five locations into two mega environments which fit well with the assignment of five locations into two corn belts widely adopted by maize breeders and grain corn growers. The above analyses revealed the high similarity in a growth environment and in PH and EH phenotype within a corn belt and large divergences in growth environment and PH and EH phenotype between the two corn belts. The correlation coefficients between PH and EH (PH*EH) within each location range from 0.51 to 0.75 (**Table 1**), which is significant at 0.001 level.

To verify the results of environmental grouping, variance analysis was conducted to reveal the differences between mega environments (**Table S2**). The results showed that there were significant genotype x mega environment interactions in both PH and EH. Genotype x mega environments accounted for 30.7% and 31.2% of the total variance for PH and EH respectively. Whereas genotype variance accounted for 32.2% and 29.2% of the total variance for PH and EH, respectively. Therefore, genotype x mega environments interaction is a very important factor in determining the phenotypic plasticity observed in PH and EH.

### Characteristics of genotype data, linkage disequilibrium and population structure

Among the 876,297 SNPs collected from 203 inbred lines, 73,174 high-quality SNP loci after a filtering procedure (see Material and Methods) were kept for all analyses in this project. The minor allele frequency (MAF) distribution (see **Figure S1A**) indicates the existence of abundant allelic polymorphism for genome-wide marker-trait association. About 60% of SNPs with heterozygosity less than 5% are only suitable to additive allelic effect analysis (see **Figure S1B**), the other 40% of SNPs with heterozygosity higher than 5% are suitable to both additive and dominant allelic effect analysis. The LD decay across all 10 chromosomes reached down to r^2^ = 0.1 when the distance between two adjacent SNP increased up to 60 kb (**Figure S2A**). The population structure analysis showed that the delta K value reached the peak at K=3, indicating that this diversity panel of 203 inbreds can be divided into three subgroups (**Figure S2B**), namely, M-Reid+P, SS+Iodent+Lan, and LRC+TSPT, respectively (**Figure S2C**).

### Identification of significant QEIs and the patterns of QTN x E interactions

12 significant QEIs for PH and 11 significant QEIs for EH were identified and reported in **Table 2** and they are visualized as pink dots on the Manhattan plots (**Figure S3A, B**), 9 of 12 QEIs for PH and 8 of 11 QEIs for EH are QEIs with additive effect as a key effect, whereas 3 of 12 QEIs for PH and 3 of 11 QEIs for EH are QEIs with dominant effect as a key effect. *S3_224* and *S10_135* are two QEIs for EH with the largest LOD (QE) and variance.

**Table 2:** QEIs between two mega-environmental groups and associated candidate genes for PH and EH.

Trait	Marker (V4, abbr)	Chr#	Position (V4, bp)	Ref/Alt	LOD (QE)	Add*E1	Dom*E1	Add*E2	Dom* E2	Var	r2 (%)	Het.	|dom|/|add|	Key effect	Gene ID	Gene Symbol	Category
PH	S1_185	1	184855257	G/A	7.0	3.1	4.8	-3.1	-4.8	10.7	2.6	0.09	1.57	add	Zm00001d031277,Zm00001d031278	*ZAT3/DOF1.6*	Upstream
	S2_85	2	85448512	A/C	10.1	-4.4		4.4		19.3	4.6	0.12	0.00	add	Zm00001d004132	*cl36164_1*	UTR5
	S2_237	2	236504893	G/A	8.1	1.9	11.1	-1.9	-11.1	13.1	3.1	0.08	5.85	dom	Zm00001d007630	*RPS2*	Non-syn.
	S3_156	3	155997977	A/G	9.3	0.2	-7.2	-0.2	7.2	14.5	3.5	0.28	29.39	dom	Zm00001d042199	*PSB28*	Syn.
	S3_159	3	158641942	A/C	6.6	3.4	6.4	-3.4	-6.4	11.9	2.9	0.02	1.90	add	–		Intergenic
	S4_40	4	40463790	T/C	11.5	-4.5	1.4	4.5	-1.4	19.8	4.7	0.02	0.30	add	Zm00001d049691,Zm00001d049692	*mads32*	Syn.
	S6_66	6	66264336	G/A	6.7	-3.4	-3.2	3.4	3.2	11.5	2.7	0.08	0.94	add	Zm00001d036014	*E3/UBPL*	Intronic
	S6_133	6	133125635	A/G	16.6	-5.5	-3.4	5.5	3.4	28.6	6.8	0.11	0.62	add	Zm00001d037655	*-*	Non-syn.
	S7_48	7	47993521	C/G	10.3	-4.1		4.1		16.8	4.0	0.11	0.00	add	Zm00001d019648	*nbp1*	Syn.
	S8_7	8	7205104	T/G	9.1	0.3	7.7	-0.3	-7.7	14.1	3.4	0.23	24.27	dom	Zm00001d008396	*-*	UTR5
	S10_149	10	148903473	C/T	13.5	6.4	1.1	-6.4	-1.1	21.6	5.2	0.49	0.17	add	Zm00001d026606	*cdj5*	Non-syn.
EH	*S1_33*	1	32857527	G/T	11.5	-3.1	-1.7	3.1	1.7	7.0	4.4	0.35	0.55	add	*Zm00001d028386*		Downstream
	*S1_86*	1	86353115	G/A	5.7	-2.3	0.6	2.3	-0.6	2.9	1.9	0.46	0.28	add	*Zm00001d029772*	*prh126*	Non-syn.
	*S1_283*	1	283402157	A/C	7.7	2.3	-1.9	-2.3	1.9	5.1	3.2	0.01	0.85	add	*Zm00001d034076*	*mmp165*	Non-syn.
	*S2_2*	2	1669905	T/C	8.7	-1.4	-3.8	1.4	3.8	4.9	3.1	0.24	2.63	dom	*Zm00001d001837*	*myb133*	Non-syn.
	*S3_94*	3	94315573	C/A	7.0	-2.5	0.7	2.5	-0.7	3.9	2.5	0.41	0.29	add	*Zm00001d041064*	*NUP1*	Non-syn.
	*S3_224*	3	223519980	C/T	17.3	3.3	1.5	-3.3	-1.5	10.3	6.5	0.04	0.44	add	*Zm00001d044272*	*bhlh94*	UTR5
	*S4_38*	4	37703788	A/G	5.8	-1.9	4.9	1.9	-4.9	3.7	2.3	0.01	2.57	dom	*Zm00001d049616*	*gpat9*	Syn.
	*S4_225*	4	224650169	T/C	14.2	3.6	-0.1	-3.6	0.1	8.1	5.1	0.36	0.01	add	*-*	*-*	Intergenic
	*S5_1*	5	1080954	T/C	7.6	-2.6	-0.3	2.6	0.3	4.9	3.1	0.27	0.10	add	*Zm00001d012848*	*-*	Non-syn.
	*S5_215*	5	214720899	A/C	10.3	3.0	0.7	-3.0	-0.7	5.6	3.6	0.41	0.23	add	*Zm00001d018122*	*E3/UBPL*	Non-syn.
	*S8_174*	8	174327122	C/A	5.0	-1.2	2.4	1.2	-2.4	2.7	1.7	0.29	2.07	dom	*Zm00001d012428*	*-*	Non-syn.
	*S10_135*	10	134518892	G/C	20.9	3.4	3.4	-3.4	-3.4	11.8	7.4	0.03	0.99	add	*Zm00001d025947*	*saur76*	Intergenic

EH, ear height; PH, plant height; LOD(QE), LOD score for QEIs; Add*E1, additive effect of E1(Summer Corn Belt); Dom*E1, dominant effect of E1(Summer Corn Belt); Add*E2, additive effect of E2(Spring Corn Belt); Dom*E2, dominant effect of E2(Spring Corn Belt); Var, the variance of each QTN; Het., proportion heterozygous; |dom|/|add|, namely |dom*E1|/|add*E1| or |dom*E2|/|add*E2|; Key effect: if |dom|/|add|≤2, or Proportion Heterozygous >0.05, Key effect would be add; if |dom|/|add|>2, and Proportion Heterozygous>0.05, Key effect would be dom. Category: location of SNPs in genes and effect, upsteam, downstream, UTR5, intergenic, intronic represent SNP locate the region of the candidate gene, Non-syn.(non-synonymous) represent the SNP locate in the exonic region of the candidate genes which cause an amino acid change, Whereas syn.(synonymous) represent the SNP locate in the exonic region of the candidate genes which do not cause an amino acid change.

**Figure 2:**
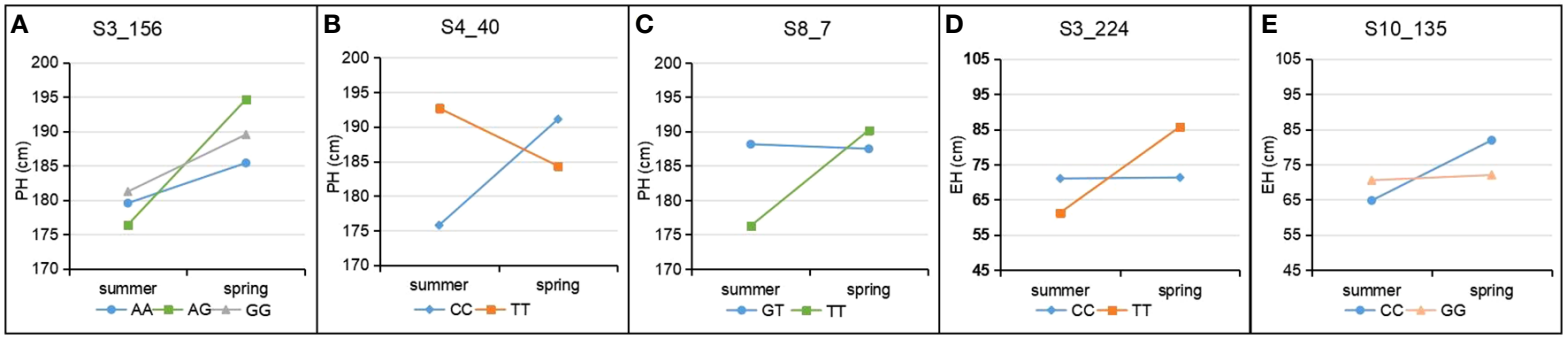
Patterns of QTN x E interaction in Summer and Spring Corn Belts for PH and EH. **(A–C)** three QEIs S3_156 **(A)**, S4_40 **(B)** and S8_7 **(C)** for PH; **(D, E)** two QEIs S3_224 **(D)** and S10_135 **(E)** for EH.

To visualize and verify the QTN x environment interaction in QEIs identified from IIIVmrMLM graphically, the patterns of QTN x environment interaction of five QEIs from **Table 2** were shown by line chart (**Figure 2**). The QTN x environment interaction was further partitioned into add*E and dom*E as shown in **Table 2**. *S3_156* is a QEI for PH with large negative dom (dominance)*E1 interaction (-7.2) at E1(Summer Corn Belt) locations and large positive dom (dominance)*E2 interaction (7.2) at E2 (Spring Corn Belt) locations, and with an absolute dom/add ratio of 29.39, **Figure 2A** illustrates the interaction pattern of its three genotypes and shows that heterozygotic AG genotype has significantly shorter PH than both “AA” and “GG” genotype at Summer Corn Belt (E1), but has much taller PH at Spring Corn Belt (E2). Another QEI with a dominant effect as key effect is S8_7 for PH (**Figures 2C**), with a high absolute dom/add ratio of 24.27. The QEIs *S4_40* for PH and *S3_224* for EH are QEIs with additive effect as key effect and absolute dom/add ratio of 0.3 and 0.44, respectively (**Table 2**), the genotype CC and TT show opposite phenotype performance in the Summer and Spring Corn Belts (**Figures 2B, D**). The QEI S10_135 has an absolute dom/add ratio of 0.99 (**Table 2**), indicating a nearly equal amount of dom*E and add*E interaction (**Table 2**; **Figure 2E**). The candidate genes for *S3_224* and *S10_135* are *Zm00001d044272* (*bhlh94*, bHLH-transcription factor 94) and *Zm00001d025947* (*saur76*, small auxin up RNA76), respectively. The candidate genes for *S4_40* are *Zm00001d049691*(*SDH6*, Succinate dehydrogenase subunit 6 mitochondrial) and *Zm00001d049692* (*MADS32*, MADS-transcription factor 32), likely an important QEI for PH.

### Identification of significant corn-belt-specific QTNs and annotations

**Figure 3:**
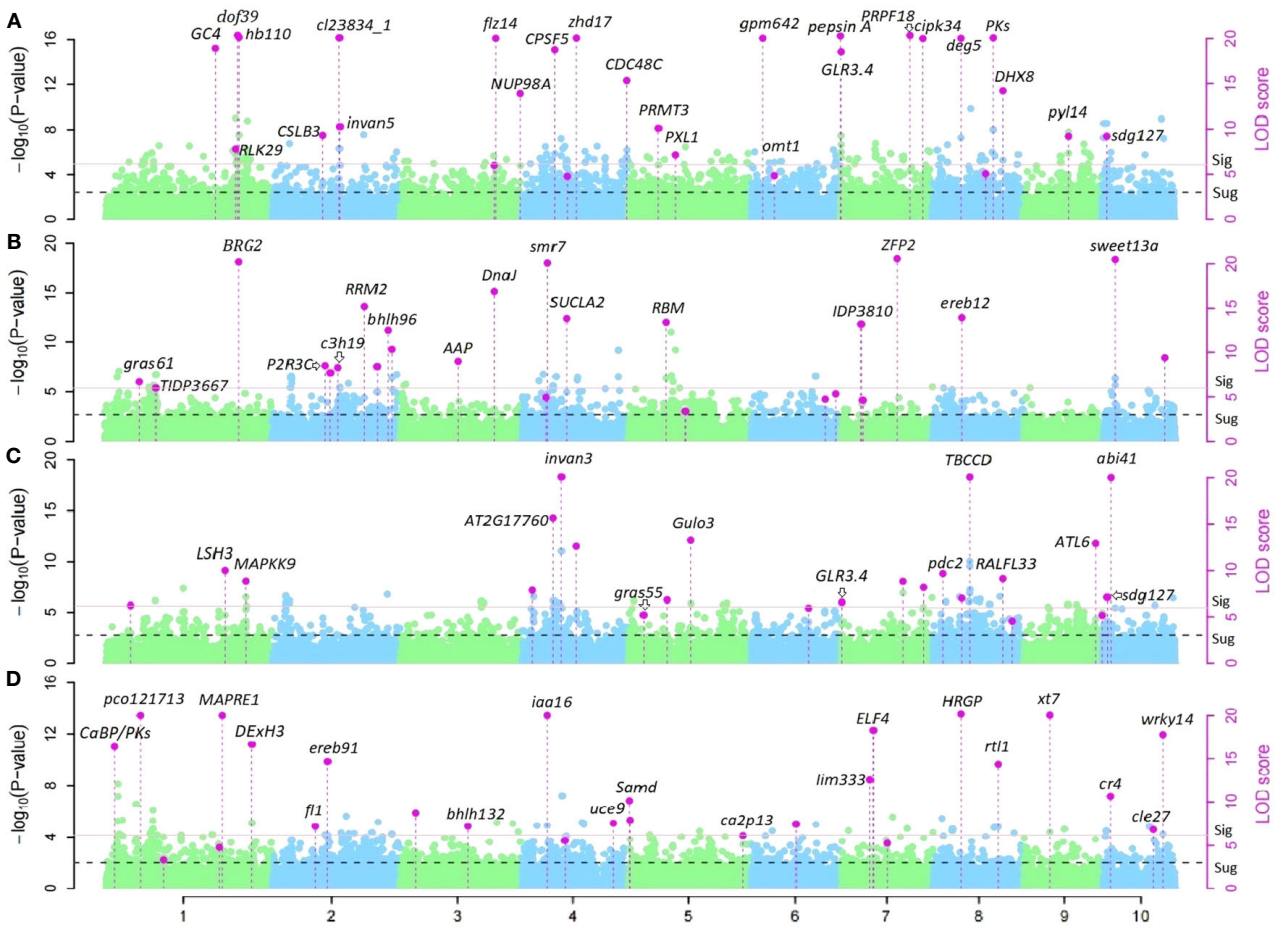
Manhattan plots of corn-belt-specific QTNs for PH and EH in Summer and Spring Corn Belt. **(A,B)** corn-belt-specific QTNs and candidate genes for PH in Summer Corn Belt **(A)** and Spring Corn Belt **(B)**; **(C, D)** corn-belt-specific QTNs and candidate genes for EH in Summer Corn Belt **(C)** and Spring Corn Belt **(D)**.

28 and 23 QTNs for PH and EH respectively were identified from Summer Corn Belt data, thus are called summer-corn-belt-specific QTNs (**Table S3**; **Figure 3**). 25 and 26 QTNs for PH and EH respectively were identified within the Spring Corn Belt, and thus are called spring corn belt specific QTNs. Among the total 102 corn-belt specific QTNs reported in **Table S3**, 56 QTNs show an additive effect as key effect (|dom/add|<2.0) and 46 QTNs show a dominant effect as key effect (|dom/add|>2.0).

QTN *S10_4* (*Zm00001d023333*, *sdg127*, set domain gene127) and *S7_1* (*Zm00001d018614*, *GLR3.4*: glutamate receptor 3.4 or *Zm00001d018616*, DDRGK domain-containing protein) are two significant summer corn belt specific QTNs for both PH and EH (**Tables 3**, **S3**). There are a set of candidate genes located within 7.0 Mb region of chromosome 1, near the three summer corn belt specific QTNs *S1_255*, *S1_259*, and *S1_262*; *Zm00001d033319* (V4:chr1:258878226:258879592, Auxin-responsive protein *IAA4*) is located 200kb from *S1_259* (V4:chr1:259066746) and *Zm00001d033369* (V4:chr1:260633725:260634703, Gibberellin-regulated protein 1) is located between *S1_259* and *S1_262* (Teale et al., 2006; Wang et al., 2017; Luo et al., 2018; Wang and Wang, 2022b; Wu et al., 2023). Another spring corn belt specific QTN, *S1_263* (V4: chr1:262565751) is also located in this region. QTN *S1_255*, *S1_259*, and *S1_262* have additive effects as key effects in the Summer Corn Belt, and the QTN *S1_263* has a dominant effect as key effect in the Spring Corn Belt (**Tables 3**, **S3**).

**Table 3:** Corn-belt-specific QTNs for PH and EH in Summer and Spring Corn Belt.

Trait	Corn belt	Marker (V4, abbr)	Chr#	Position (V4, bp)	LOD (Q)	Add	Dom	Var	r2 (%)	Het.	|dom|/|add|	Key effect	Gene ID	Gene Symbol	Category
PH	E1	S1_255	1	255244221	7.8	2.5	0.9	5.6	1.0	0.05	0.37	add	Zm00001d033230	RLK29	Non-syn.
PH	E1	S1_259	1	259066746	55.8	7.3	-0.1	21.6	3.8	0.06	0.01	add	Zm00001d033325	dof39	upstream
PH	E1	S7_1	7	910582	18.5	4.2	-4.8	17.9	3.1	0.06	1.15	add	Zm00001d018614	GLR3.4	Non-syn.
PH	E1	S10_4	10	3618262	9.2	2.9	2.6	8.3	1.4	0.06	0.88	add	Zm00001d023333	sdg127	Non-syn.
PH	E2	S7_151	7	150642747	76.4	17.2	2.1	34.7	3.0	0.12	0.12	add	Zm00001d021386	ZFP2	Non-syn.
PH	E2	S10_15	10	15032123	67.9	7.0	16.4	19.3	1.7	0.73	2.34	dom	Zm00001d023677	sweet13a	Syn.
EH	E1	S1_273	1	273051629	8.8	2.2	-0.3	4.3	2.1	0.07	0.13	add	Zm00001d033765	MAPKK9	upstream
EH	E1	S4_118	4	117960613	29.5	-3.9	-1.0	9.5	4.8	0.01	0.25	add	Zm00001d050715, Zm00001d050716	invan3	upstream
EH	E1	S7_1	7	1024439	6.6	-1.6	1.6	2.5	1.2	0.06	0.97	add	Zm00001d018615	GLR3.4	Non-syn.
EH	E1	S10_4	10	3618262	7.1	1.9	-0.1	3.5	1.7	0.06	0.07	add	Zm00001d023333	sdg127	Non-syn.
EH	E2	S1_7	1	7065140	16.4	-0.9	7.9	15.3	3.0	0.28	8.98	dom	Zm00001d027503,Zm00001d027508	CaBP/PKs	Non-syn.
EH	E2	S4_41	4	41323782	21.1	5.3		10.2	2.0	0	0	add	Zm00001d049715,Zm00001d049717	iaa16	Syn.

the abbreviation in this table is same as **Table 1** and **2**. |dom|/|add|: the absolute ratio of dominant effect to additive effect.

### Candidate genes association mapping and tissue-specific expression analysis

**Figure 4:**
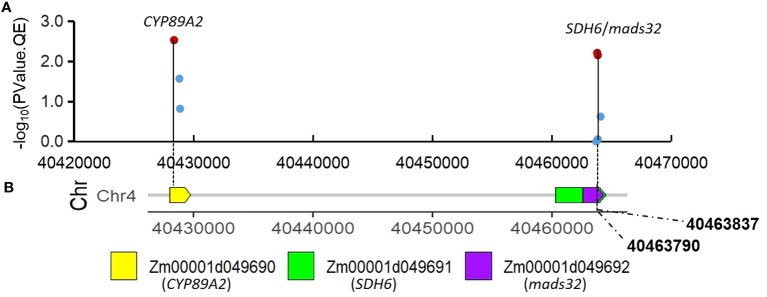
Association of SNPs surrounding significant QEI *S4_40* with candidate genes. **(A)** associations of the twelve SNPs using mean value of PH in Summer and Spring Corn Belt; **(B)** gene distribution around *S4_40*(V4:chr4:40463790).

Candidate gene search has found that the significant QEI *S3_224* identified by 3VmrMLM is located on the 5’UTR region of *Zm00001d044272* (*bhlh94*), its gene structure is shown in **Figure S4**. Another QEI, *S4_40* (full ID: S4_40463790, V4: chr4:40463790) is on the exon of two partially overlapping candidate genes *Zm00001d049691*(V4:chr4:40460274 - 40464504) and *Zm00001d049692* (chr4:40462578 - 40464305) (**Figure 4**. **Tables 2**, **S4**). Tissue-specific expression analysis shows *Zm00001d049691*(*SDH6*) expresses in stems, leaves, embryos, roots, spikelets, and silks, *Zm00001d049692* (*MADS32*) expresses in stems, splikelets, and silks, and *Zm00001d049690* (*CY P89A2*) only expresses in roots (**Figure S5**). *SDH* encodes succinate dehydrogenase, which is activated by salt stress (Fedorin et al., 2023) and is also regulated by light (Eprintsev et al., 2016). Another MADS-transcription factors, *ZmMADS4* and *ZmMADS67* both increase leaf number and delayed flowering, indicating that they promote the floral transition (Sun et al., 2020) and overexpression of *ZmMADS69* causes early flowering (Liang et al., 2019).

**Figure 5:**
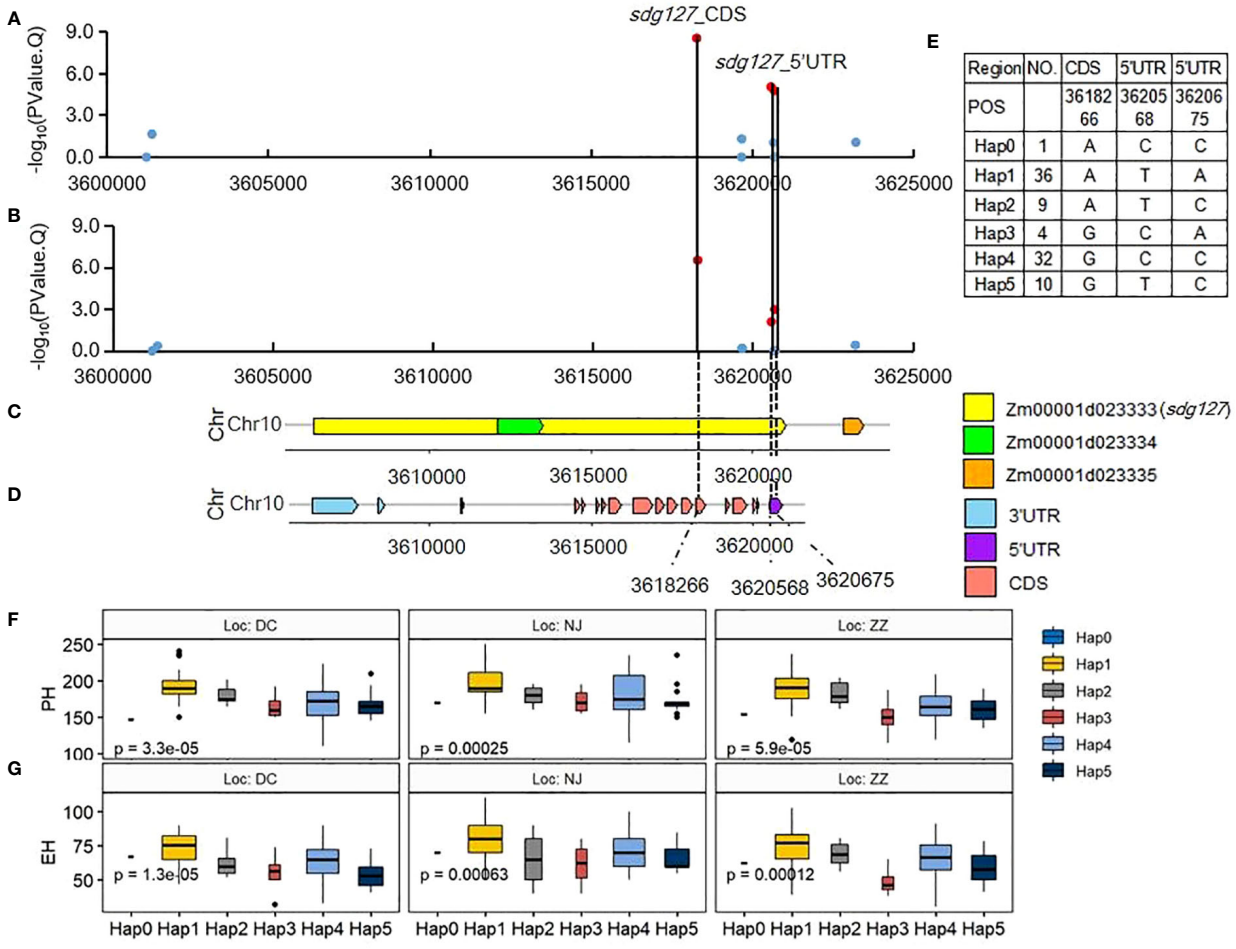
Association of SNPs surrounding significant QTN *S10_4* with candidate genes and their haplotype Effects. **(A, B)** associations of the 11 SNPs with PH **(A)** and EH **(B)** in Summer Corn Belt. The dot is red with the threshold of -log10(PValue)>2; **(C)** gene distribution around QTN *S10_4*(V4:chr10:3618266); **(D)** gene structure of *Zm00001d023333*; **(E)** haplotypes of the three significant SNPs; **(F, G)** boxplots of haplotypes for PH **(F)** and EH **(G)** in Summer Corn Belt.

Three SNPs surrounding QTN *S10_4* located in *Zm00001d023333* are significant at 0.01 level (-log10-P >2) for PH and EH in the Summer Corn Belt (**Figures 5A, B**). Two of them: the S10_3620568 and S10_3620675 are located on 5’UTR and the S10_3618266 is located on CDS (**Figures 5C, D**). *Zm00001d023333* (Chr10:3606398-3621010, *sdg127*, SET domain gene127) encodes a histone-lysine N-methyltransferase ATXR7. Another two SET domain family genes, SET domain group 8 (*SDG 8*) in *Arabidopsis thaliana* (Zhao et al., 2005) and *SDG712* in rice (Zhang et al., 2021) could delay flowering by repressing the expression of FLOWERING LOCUS C (FLC) and florigen genes, respectively. The above research findings suggest that *Zm00001d023333* we identified in this study might affect PH and EH by delaying flowering time and lengthening vegetative growth. Haplotype analysis has shown that the three SNPs can form six haplotypes (Hap0, Hap1, Hap2, Hap3, Hap4, Hap5) (**Figure 5E**). Hap 1 (ATA) and Hap 4(GCC) are the major haplotypes, with 36 and 32 inbreds, respectively. Hap 1 (ATA) is higher than Hap 4(GCC) in terms of both PH and EH (**Figures 5F, G**).

Several SNPs significantly associated with PH and EH are identified surrounding QTN locus *S7_1*. Some of them are located on the CDS of the two candidate genes *Zm00001d018614* and *Zm00001d018616*. Expression of *Zm00001d018616* (about 30 FPKM) at the mRNA level is ten times higher than *Zm00001d018614* (about 3 FPKM) in the stem (**Figure S6**). *Zm00001d018614* and *Zm00001d018615* are genes encoding glutamate receptor, which are involved in seed germination inhibition and seedling heat tolerance (Kong et al., 2015; Li et al., 2019). Another candidate gene, *Zm00001d018617* (*ga2ox12*, gibberellin 2-oxidase12, Chr7:1105512-1106576), is a member of gibberellin oxidase gene family which might affect PH (Paciorek et al., 2022), but its expression is not detected in stem tissues of maize (**Figure S6**).

Three SNPs associated with PH are identified surrounding QTN *S10_15*, a spring-corn belt specific QTN and they are all located in the CDS region of candidate gene *Zm00001d023677* (*sweet13a*, V4:chr10:15030181-15032801) (**Figure S7**); two SNPs, S10_15032123 and S10_15032153, are synonymous SNV whereas the third SNP, S10_15032160, is nonsynonymous SNV which causes an amino acid change (**Table S5**). Haplotype analysis has shown that the three SNPs can form four haplotypes (H1, H2, H3, H4). The PH of heterozygous haplotype H2 (CG/CG/TG) is significantly higher than that of the homozygous haplotype H2 (CC/GG/GG) (**Figure S7**). The candidate gene *Zm00001d023677* (*sweet13a*) encodes a SWEET protein of the MtN3/saliva family (Xuan et al., 2013). Another SWEET protein coding gene *CmSWEET17*, has been reported to be involved in the process of sucrose-induced axillary bud outgrowth in strawberry (*C. morifolium*), possibly via the auxin transport pathway (Liu et al., 2020a).

## Discussion

### Mega environment, phenotypic plasticity, and mega-environmental GEI and QEI

Partitioning multi-environments into a set of environment clusters or mega environments has been well-studied in which, the multi-environments were grouped using PCA, clustering, and GGE biplot (Shu and Fan, 1986; Yan and Kang, 2003). Yan (2015) defined a mega-environment as a group of geographical environments that share the same (sets of) genotypes consistently across years. Other researchers have defined a mega-environment as a group of growing environments that are similar in terms of genotype response and that show a repeatable relative performance of a set of crop genotypes across years (Yan and Rajcan, 2002). Mega-environments are often identified through the analysis of multiple-environment trial data for a set of genotypes. The purpose of the mega-environment analysis is to understand the nature of environmental variation across experimental locations, whether there is structure or segmentation among the locations. Our result shows that there is significant segmentation among the 5 locations and they can be divided into two mega-environments, there is very little variation among locations within a mega environment and the two segments fall right into the two corn belts that have been widely adopted by breeders and corn growers. Our results also show that the GGE model, with a biplot display, is an effective tool for displaying environment structure and segmentation which explain why it has become popular in analyzing multiple-environment trial data to determine environment cluster (Yan and Kang, 2003; Yan et al., 2011; Yan, 2015; Dai et al., 2010).

Understanding the genetic basis of phenotypic plasticity in general and the genotype x environment interaction (GEI) in particular is of primary importance in traditional crop genetics and plant breeding, and a large body of literature on models and strategies is available (Shu and Fan, 1986; Cooper and DeLacy, 1994; Malosetti et al., 2013; Li et al., 2018; Liu et al., 2020b; Schneider, 2022). The genetic bases of genotype x environment interaction (GEI) for PH and EH are difficult to study due to environment structure and segmentation among experiment locations and the multi-locus nature of their genetic control. In this study, we deal with multi-environmental segmentation by grouping multiple locations into mega-environments using GGE biplot and deal with multi-locus nature by dissecting it into QTN x environment interaction or QEIs using multi-environmental GWAS. Our results show that genotype x mega environment interaction (GEI) accounted for about 30% of the total variation for both PH and EH, almost equal to the genotypic variation among 203 inbred lines in proportion (which is also about 30%). Therefore, genotype x mega environments interaction has a significant contribution to the phenotypic plasticity observed in PH and EH.

Understanding the molecular mechanism underlying the detected pattern of phenotypic plasticity in general and G x E, in particular, has been a major effort in the last decade. QTL mapping and genome-wide association studies (GWAS) have been shown effective means in identifying a large number of QTL/QTN and QEIs (Xiao et al., 2017; Jin et al., 2023; Napier et al., 2023) and transcriptomic analysis and functional genomics have been shown as important ways to identify candidate genes and verify their biological functions (Seyfferth et al., 2021; Han et al., 2023; Napier et al., 2023; Wang et al., 2023). Various statistical models and bioinformatic algorithms have been proposed to improve the effectiveness of GWAS but no significant progress has been made on GWAS that can partition GEI and identify QEIs. We have shown that the 3VmrMLM GWAS models and the IIIVmrMLM software package recently released can effectively identify QEIs. The software package has also been applied to data from rice, soybean, and other crops to identify QEIs and hunt candidate genes underlying QEIs (Zhang et al., 2022; Zuo et al., 2022; Zhao et al., 2023). We have shown that by employing 3VmrMLM multi**-**environment GWAS models, we were able to go beyond the traditional G x E interaction analysis and were able to identify and annotate a set of QEIs for PH and EH.

Among the candidate genes annotated by transcriptomic analysis, *Zm00001d049692* (*MADS32*) surrounding QEI *S4_40*, might affect PH in different ecological zones by both increasing leaf number, delay flowering time, and lengthen vegetative growth period, similar to *ZmMADS4* and *ZmMADS67* (Sun et al., 2020). *Zm00001d044272* (*bhlh94*) surrounding QEI *S3_224* might be involved in low-temperature responsiveness, MeJA-responsiveness, abscisic acid responsiveness because of its cis-regulatory elements and affect root growth and elongation in response to stressful conditions as the manner of RICE SALT SENSITIVE3 (RSS3) in rice (Toda et al., 2013). These findings will facilitate the understanding of the molecular basis of the G x E observed in PH and EH.

### Corn belt-specific QTNs

As has been partly described in the Material and Method section, the summer corn-belt average and spring corn-belt average were used to identify QEI, which is defined as the QTN that shows significant QTN x corn-belt interaction by IIIVmrMLM. When QTN x environment interaction is significant, the significant positive and negative genotype effects were canceled out during averaging, therefore the QTN main effects become less meaningful. We obtain corn belt specific QTNs by feeding the IIIVmrMLM software with multi-location data within a corn belt. A corn belt specific QTN is a QTN that shows a significant genotype effect within either summer or spring corn belt data. QEIs explain the phenotypic plasticity across different corn belts and are frequently the targets to select against by breeders seeking stress tolerance and trait stability whereas corn-belt specific QTNs expain the genetic variation within a corn-belt and are frequently targets to select for by breeders seeking genetic gain and stable phenotypic performance in the corresponding corn belt.

**Figure 6:**
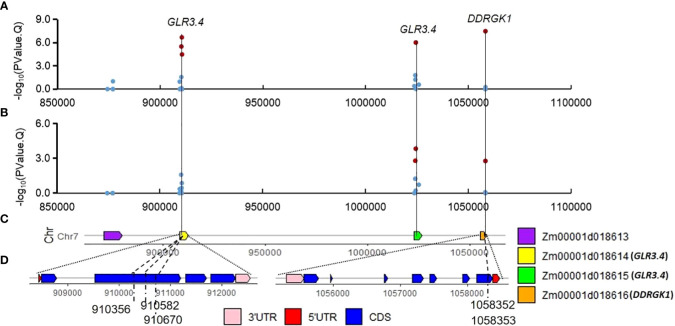
Significant QTN S7_1 and associated SNPs on candidate gene *Zm00001d018614* (*GLR3.4*) and *Zm00001d018616* (DDRGK domain-containing protein). **(A, B)** associations of the 28 SNPs for PH **(A)** and EH **(B)** in Summer Corn Belt; **(C)** gene distribution around S7_1 (V4:chr7:910582); **(D, E)** gene structure of *Zm00001d018614* **(D)** and *Zm00001d018616* **(E)**.

We have identified a set of main effect QTNs or corn belt specific QTNs. In the Summer Corn Belt, four candidate genes *Zm00001d018614*, *Zm00001d018615*, *Zm00001d018616*, and *Zm00001d018617* are identified surrounding QTNs *S7_1* (**Figures 6**, **S6**). *Zm00001d018617* is also identified by Zhang et al. (2019) as a candidate gene for PH. *Zm00001d033230* surrounding QTN S1_255 (V4:chr1: 255244221, **Tables 3**, **S3**; **Figure 3**) is associated with PH in the Summer Corn Belt in our study, which is also identified as a candidate gene associated with PH in Zmdle1, a dwarf and low ear maize mutant (Zhou et al., 2023). *Zm00001d049715* (*IAA25*) surrounding QTN *S4_41* is associated with EH in the Spring Corn Belt, which is also identified as a candidate gene for PH by Zheng et al. (2016) through meta-QTL analysis.

### 3VmrMLM multi-environment GWAS models

The selection of appropriate statistical models to detect and measure association is critical to the success of GWAS. The models should be able to deal with various features of phenotypic and genotype data, such as continuity and normality of phenotypic data, population structure and kinship in genotype data, and various confoundings from other covariables in a model. The R software package provided by Zhang’s group, IIIVmrMLM V1.0 (Li et al., 2022a; Li et al., 2022b), is a GWAS model that fits the data of strong G x E. Under the framework of a compressed variance component mixed model, each marker on the maize chromosome was first scanned for statistical significance and a less stringent Banforroni correction was adopted in the statistical test and the significant marker loci identified were then incorporated into a new multi-locus genetic model and their effects were estimated by Empirical Bays and all non-zero effects were further evaluated by the likelihood ratio test. Another feature of the 3VmrMLM model is that it can take advantage of heterozygosity discovered in genomic sequence data. Heterozygosity has been detected in many DNA sequence projects in corn inbred lines that have been selfed for 6-10 generations, Traditionally, this so-called residual heterozygosity is treated as sequencing errors, or as missing data and is filtered out and ignored. The recent hi-fi sequencing technology has shown this heterozygosity is not a sequencing error and is instead a true variation in inbred lines. The 3VmrMLM model can utilize this important information to reveal QTN x QTN and QTN x environment interaction.

## Data availability statement

The datasets presented in this study can be found in online repositories. The names of the repository/repositories and accession number(s) can be found below: European Variation Archive (EVA) at EMBL-EBI, accession number is PRJEB64281 (The European Bioinformatics Institute< EMBL-EBI).

## Author contributions

GS: Writing – original draft, Writing – review & editing. AifangW: Writing – review & editing. XW: Data curation, Investigation, Writing – original draft. RC: Validation, Writing – review & editing. FG: Validation, Writing – review & editing. AifenW: Writing – review & editing. TL: Data curation, Writing – review & editing. YW: Funding acquisition, Supervision, Writing – review & editing.

## Supplementary material

Supplementary Figure 1 Frequency distribution of minor allele and proportion of heterozygous genotypes in 203 maize inbred lines based on 73175 SNPs dataset. **(A)** minor allele frequency; **(B)** proportion of heterozygous genotypes.

Supplementary Figure 2 Linkage disequilibrium decay and genetic diversity in the genome-wide association study (GWAS) panel. **(A)** linkage disequilibrium decay across all 10 maize chromosomes; **(B)** the plot of delta K; **(C)** population structure of the 203 lines at K = 3.

Supplementary Figure 3 Manhattan Plot of QEIs and associated known candidate genes. **(A)** QEIs and their associated genes for PH identified from mean values of PH in Summer Corn Belt (E1) and Spring Corn Belt (E2). **(B)** QEIs and their associated genes for EH from mean values of EH in Summer Corn Belt (E1) and Spring Corn Belt (E2).

Supplementary Figure 4 Association of SNPs surrounding significant QEI *S3_224* with candidate genes. **(A)** associations of the fourteen SNPs using mean values of EH in Summer Corn Belt (E1) and Spring Corn Belt (E2). **(B)** gene structure of *Zm00001d044272*(*bhlh94*).

Supplementary Figure 5 Tissue-specific expression profiles of candidate genes around QTN *S10_4* retrieved from maizeGDB.**(A)** *Zm00001d049690* **(B)** *Zm00001d049691* **(C)** *Zm00001d049692*.

Supplementary Figure 6 Tissue-specific expression profiles of candidate genes around QTN *S7_1* retrieved from maizeGDB. **(A)** *Zm00001d018614* **(B)** *Zm00001d018615* **(C)** *Zm00001d018616* **(D)** *Zm00001d018617*.

Supplementary Figure 7 Association of SNPs surrounding significant QTN S10_15 with candidate genes and their haplotype Effects. **(A)** associations of the SNPs surrounding S10_15 for PH in Spring Corn Belt. **(B)** proportion of heterozygous genotypes of the SNPs surrounding S10_15. **(C)** gene structure of *Zm00001d023677*. **(D)** haplotypes of the three significant SNPs. **(E)** boxplots of haplotypes for PH in five locations.
