# Supplementary figures and images for: Identification of QTNs, QTN-by-environment interactions for plant height and ear height in maize multi-environment GWAS
Source: Front Plant Sci. 2023 Nov 29;14:1284403. doi: 10.3389/fpls.2023.1284403 (PMC10716222; doi:10.3389/fpls.2023.1284403)

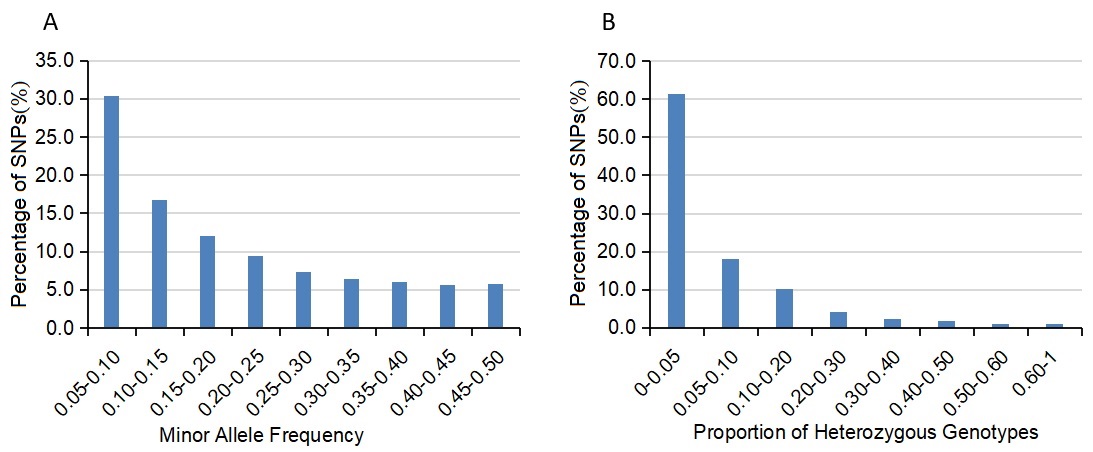

Supplement: Supplementary Figure 1 — Frequency distribution of minor allele and proportion of heterozygous genotypes in 203 maize inbred lines based on 73175 SNPs dataset. (A) minor allele frequency; (B) proportion of heterozygous genotypes. [file Image_1.jpeg]

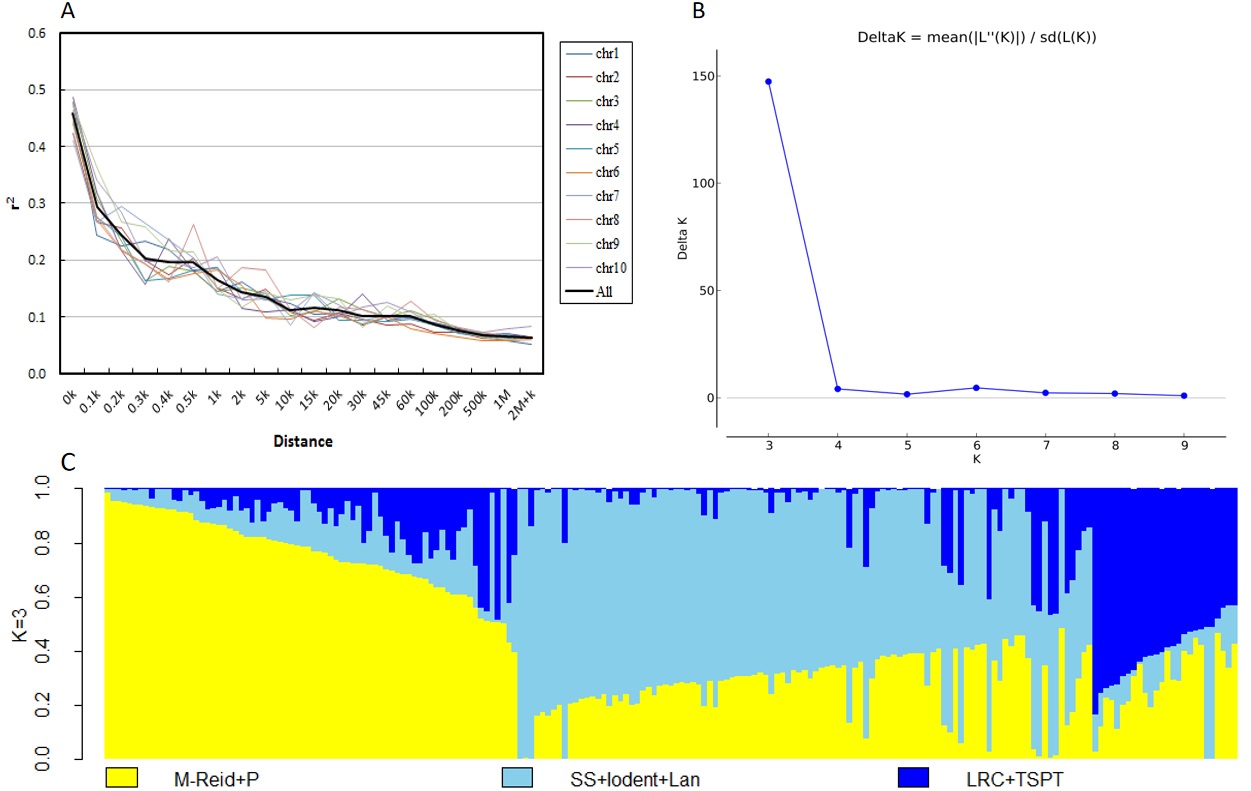

Supplement: Supplementary Figure 2 — Linkage disequilibrium decay and genetic diversity in the genome-wide association study (GWAS) panel. (A) linkage disequilibrium decay across all 10 maize chromosomes; (B) the plot of delta K; (C) population structure of the 203 lines at K = 3. [file Image_2.jpeg]

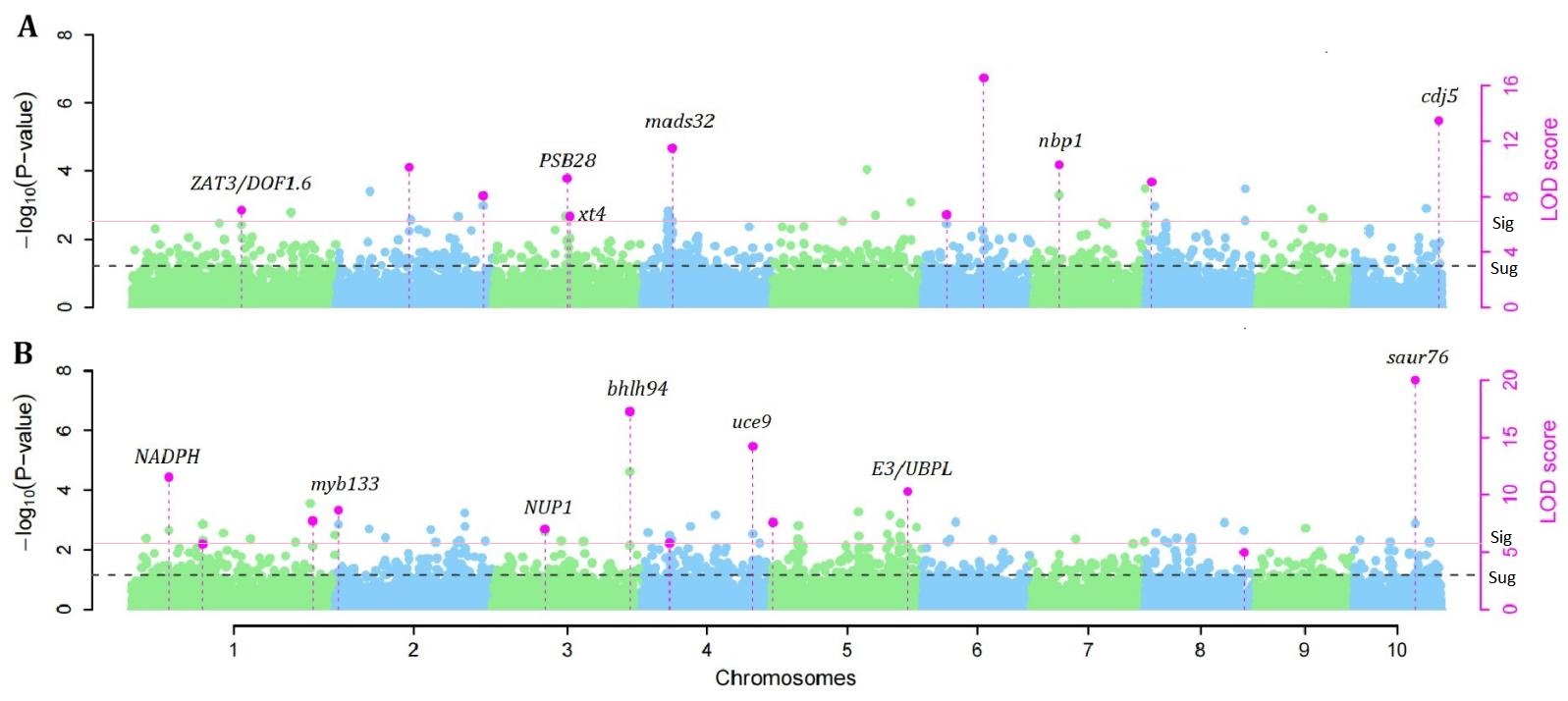

Supplement: Supplementary Figure 3 — Manhattan Plot of QEIs and associated known candidate genes. (A) QEIs and their associated genes for PH identified from mean values of PH in Summer Corn Belt (E1) and Spring Corn Belt (E2). (B) QEIs and their associated genes for EH from mean values of EH in Summer Corn Belt (E1) and Spring Corn Belt (E2). [file Image_3.jpeg]

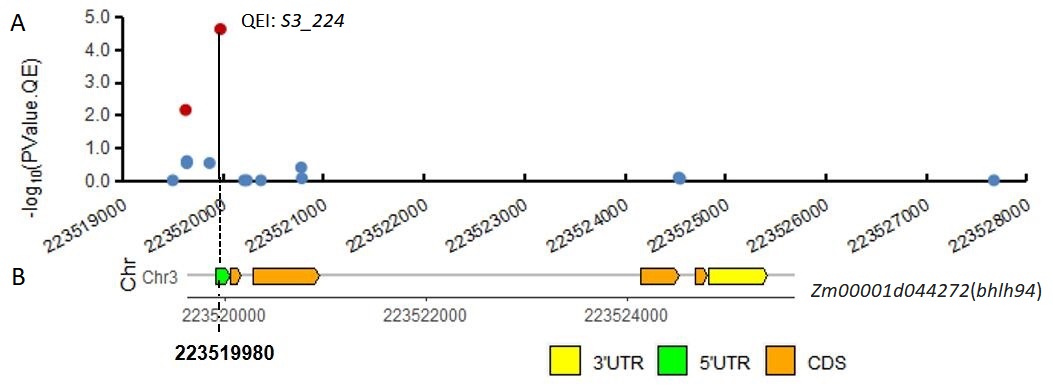

Supplement: Supplementary Figure 4 — Association of SNPs surrounding significant QEI S3_224 with candidate genes. (A) associations of the fourteen SNPs using mean values of EH in Summer Corn Belt (E1) and Spring Corn Belt (E2). (B) gene structure of Zm00001d044272(bhlh94). [file Image_4.jpeg]

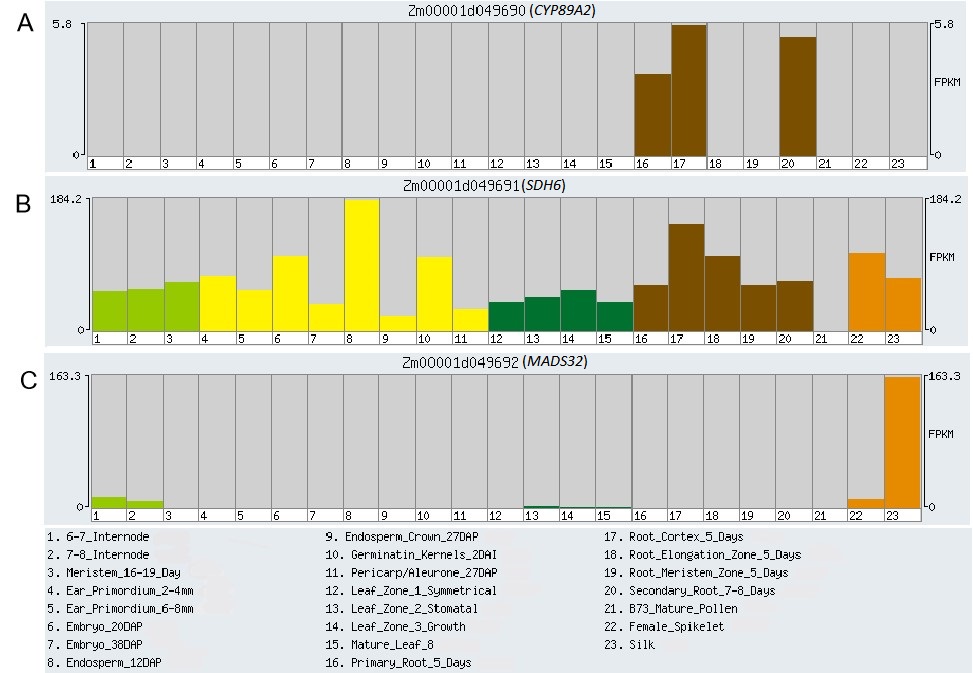

Supplement: Supplementary Figure 5 — Tissue-specific expression profiles of candidate genes around QTN S10_4 retrieved from maizeGDB.(A) Zm00001d049690 (B) Zm00001d049691 (C) Zm00001d049692. [file Image_5.jpeg]

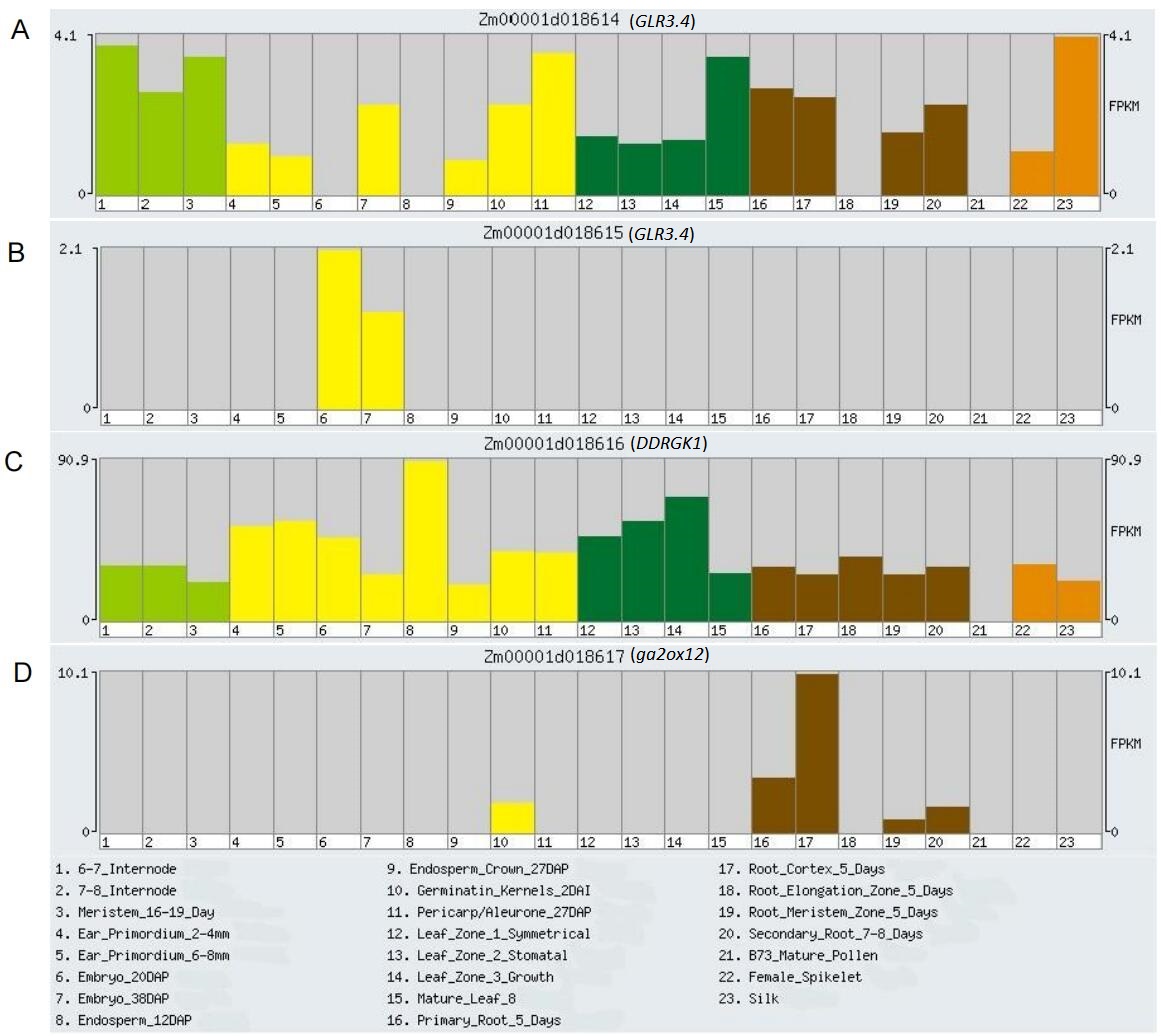

Supplement: Supplementary Figure 6 — Tissue-specific expression profiles of candidate genes around QTN S7_1 retrieved from maizeGDB. (A) Zm00001d018614 (B) Zm00001d018615 (C) Zm00001d018616 (D) Zm00001d018617. [file Image_6.jpeg]

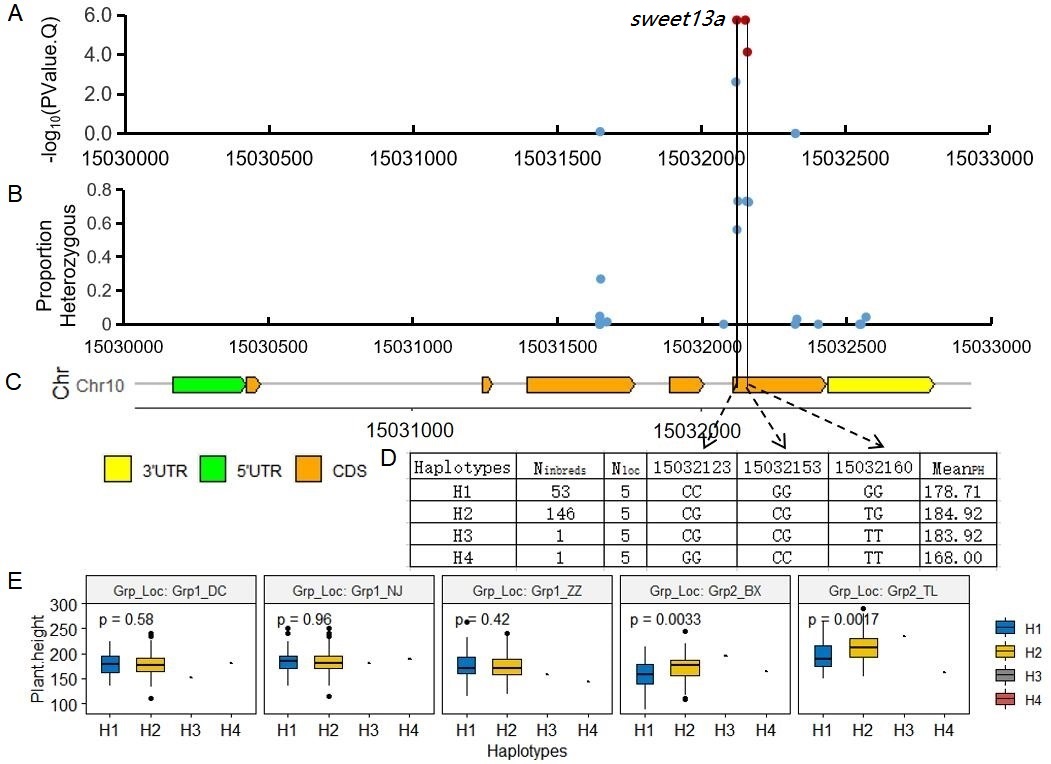

Supplement: Supplementary Figure 7 — Association of SNPs surrounding significant QTN S10_15 with candidate genes and their haplotype Effects. (A) associations of the SNPs surrounding S10_15 for PH in Spring Corn Belt. (B) proportion of heterozygous genotypes of the SNPs surrounding S10_15. (C) gene structure of Zm00001d023677. (D) haplotypes of the three significant SNPs. (E) boxplots of haplotypes for PH in five locations. [file Image_7.jpeg]
